# Predicting lymphovascular invasion in clinically node-negative breast cancer detected by abbreviated magnetic resonance imaging: Transfer learning vs. radiomics

**DOI:** 10.3389/fonc.2022.890659

**Published:** 2022-09-15

**Authors:** Bao Feng, Zhuangsheng Liu, Yu Liu, Yehang Chen, Haoyang Zhou, Enming Cui, Xiaoping Li, Xiangmeng Chen, Ronggang Li, Tianyou Yu, Ling Zhang, Wansheng Long

**Affiliations:** ^1^ Department of Radiology, Jiangmen Central Hospital, Jiangmen, Guangdong, China; ^2^ School of Electronic Information and Automation, Guilin University of Aerospace Technology, Guilin, China; ^3^ Department of Breast, Jiangmen Central Hospital, Jiangmen, Guangdong, China; ^4^ Department of Pathology, Jiangmen Central Hospital, Jiangmen, Guangdong, China; ^5^ School of Automation Science and Engineering, South China University of Technology, Guangzhou, China; ^6^ Department of Radiology, Nanfang Hospital, Southern Medical University, Guangzhou, China

**Keywords:** magnetic resonance imaging, lymph nodes, breast neoplasms, radiomic analysis, transfer learning

## Abstract

**Objective:**

To compare the performance of abbreviated breast magnetic resonance imaging (AB-MRI)-based transfer learning (TL) algorithm and radionics analysis for lymphovascular invasion (LVI) prediction in patients with clinically node-negative invasive breast cancer (IBC).

**Methods:**

Between November 2017 and October 2020, 233 clinically node-negative IBCs detected by AB-MRI were retrospectively enrolled. One hundred thirty IBCs from center 1 (37 LVI-positive and 93 LVI-negative) were assigned as the training cohort and 103 from center 2 (25 LVI-positive and 78 LVI-negative) as the validation cohort. Based on AB-MRI, a TL signature (TLS) and a radiomics signature (RS) were built with the least absolute shrinkage and selection operator (LASSO) logistic regression. Their diagnostic performances were validated and compared using areas under the receiver operating curve (AUCs), net reclassification improvement (NRI), integrated discrimination improvement (IDI), decision curve analysis (DCA), and stratification analysis. A convolutional filter visualization technique was used to map the response areas of LVI on the AB-MRI.

**Results:**

In the validation cohort, compared with RS, the TLS showed better capability in discriminating LVI-positive from LVI-negative lesions (AUC: 0.852 vs. 0.726, p < 0.001; IDI = 0.092, p < 0.001; NRI = 0.554, p < 0.001). The diagnostic performance of TLS was not affected by the menstrual state, molecular subtype, or contrast agent type (all p > 0.05). Moreover, DCA showed that the TLS added more net benefit than RS for clinical utility.

**Conclusions:**

An AB-MRI-based TLS was superior to RS for preoperative LVI prediction in patients with clinically node-negative IBC.

## Introduction

Lymphovascular invasion (LVI) is a well-recognized risk factor for disease recurrence and shorter survival in patients with invasive breast cancer (IBC), especially those with negative lymph nodes (1–3). Furthermore, it is a potential biomarker associated with chemoresistance in neoadjuvant chemotherapy (4, 5) and axillary nodal metastasis in early-stage breast cancer (6). Although these observations indicated that predicting LVI preoperatively might facilitate individualized and precise treatment for patients with IBC, the preoperative identification of LVI remains a challenge in clinical practice.

Magnetic resonance imaging (MRI), which can characterize the entire lesion with high spatial resolution, is increasingly studied with LVI assessment in IBC (7–9). However, the time consumption and high cost of the conventional breast MRI protocol hinder its broader use. Thus, a new way to increase access to breast MRI is needed. Abbreviated breast MRI (AB-MRI) is being proposed as an alternative to the full protocol because it reduces the image acquisition time, interpretation complexity, and examination costs while maintaining equivalent breast cancer detected ability (10–13). With the increasing use of AB-MRI, a large number of breast cancer were detected. Whether these breast cancers can be further staged preoperatively based on AB-MRI has attracted more and more concerns because using a one-stop imaging modality to detect and diagnose preoperative stage breast cancer would be cost-effective. Recent studies have shown that AB-MRI is effective in diagnosing breast cancer and mapping the local extent of the tumor (14, 15), and AB-MRI-based radiomics was preliminarily used for LVI assessment (16). However, the diagnostic performance was only moderate. It is needed to develop a more accurate and effective approach for LVI prediction in patients with IBC.

Radiomics is a promising tool for the characterization of breast cancer by extracting quantitative features, but the main drawbacks of using traditional radiomics analysis are time-consuming lesion segmentation and hard-coded feature extraction (17). Compared with traditional radiomics, a convolutional neural network (CNN) algorithm extracts features by using hierarchical convolution operations from the raw medical image and does not require precise tumor delineation (18). Furthermore, it has the advantage of automatically learning and hierarchically organizing task-adaptive image features, tending to reflect the high-dimensional association between images and clinical issues (19). However, the success of CNN largely depends on large training datasets (20). When the available datasets are small, transfer learning (TL) may be an alternative effective feature extraction method (21–23). Despite the convenience and advances in technology, the efficiency of a TL algorithm based on AB-MRI in predicting LVI remains unclear.

Hence, the purpose of this study was to evaluate the performance of an AB-MRI-based TL algorithm and compare it with that of radiomics for LVI prediction in patients with clinically node-negative IBC.

## Materials and methods

### Patients

**Figure 1:**
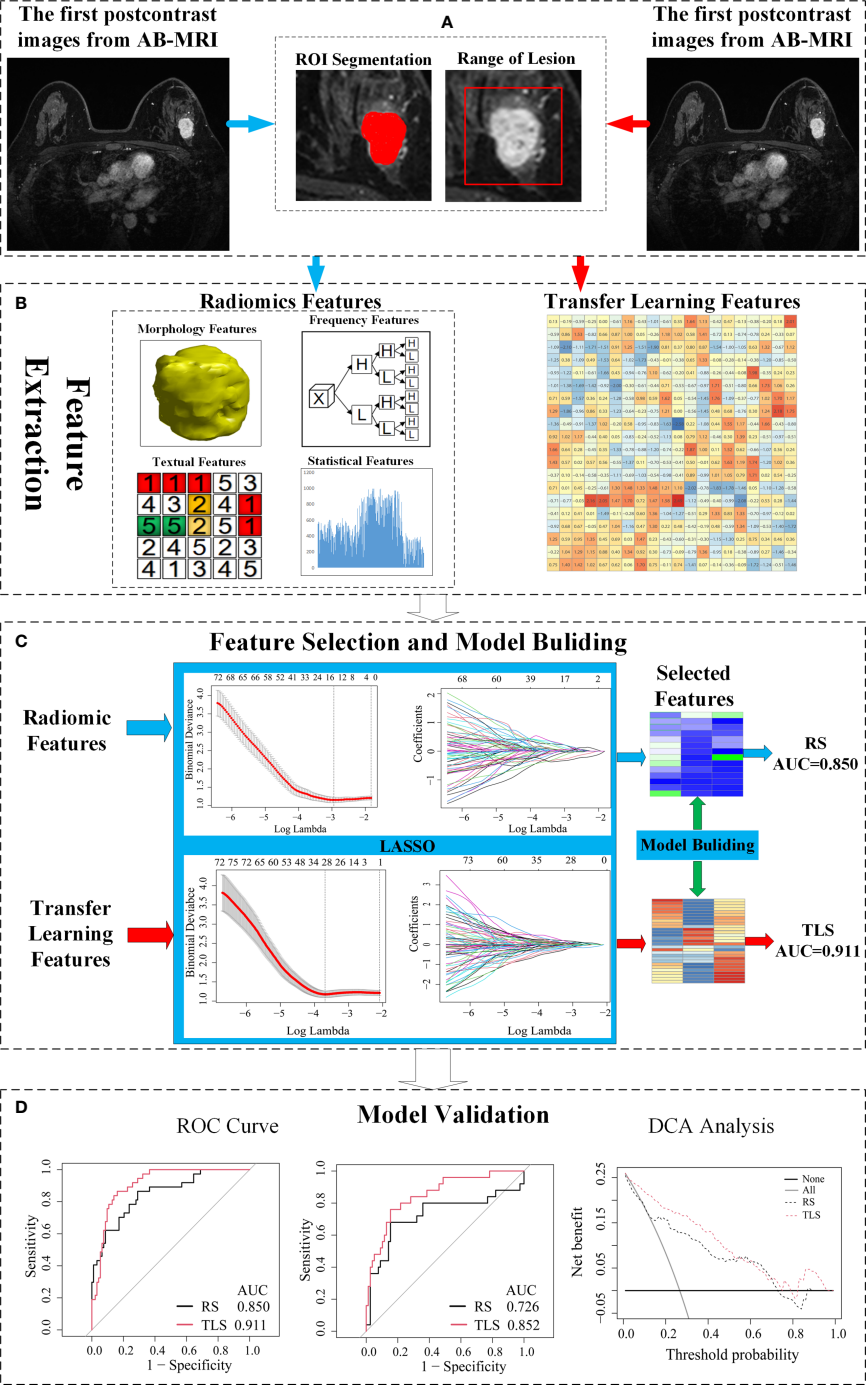
Schematic of the study design. **(A)** The image acquisition and ROI delineation, where the ROI segmentation for radiomics on the left and ROI acquisition for the transfer learning on the right. **(B)** Radiomics feature and transfer learning feature extraction. **(C)** Feature selection and a signature building. **(D)** Independent validation and comparison of models. ROI, region of interest; AB-MRI, abbreviated breast magnetic resonance imaging; LVI, lymphovascular invasion; RS, radiomics signature; TLS, transfer learning signature; ROC, receiver operating characteristics; AUC, area under curve.

A schematic illustration of the study design is presented in **Figure 1**.

The ethics committee approved this retrospective study of two participating centers with a waiver for informed consent.

Between November 2017 and October 2020, the study enrolled consecutive women with new IBC detected by AB-MRI and clinically lymph node-negative in the study. These patients underwent AB-MRI for breast cancer screening or problem resolving. The inclusion criteria were as follows: (a) underwent AB-MRI and have enhanced lesions on MR image; (b) lesions diagnosed as invasive ductal carcinoma based on pathologic evaluation of surgical specimens; (c) time interval between surgery (mastectomy or lumpectomy) and MRI examination: <2 weeks. The exclusion criteria were as follows: (a) biopsy performed before AB-MRI (n = 58); (b) received neoadjuvant chemotherapy (n = 30) or radiotherapy (n = 55) before MRI examination; (c) node-positive diagnosed on preoperative ultrasonography (n = 37): rounded hypoechoic node, complete or partial effacement of the fatty hilum, focal or diffuse cortical thickening (≥5 mm), complete or partial replacement of the node with an ill-defined or irregular mass, extra-hilar blood vessel flow on color Doppler images, or microcalcifications in the node; (d) MR image with obvious artifacts (n = 15); (e) diagnosed as benign tumors, high-risk lesions, pure ductal carcinoma *in situ* or special types of invasive breast carcinoma at final pathologic evaluation (n = 69). Finally, a total of 233 patients (age range: 30–79 years; 62 LVI-positive and 171 LVI-negative) were included. Patients from center 1 were assigned as the training cohort (37 LVI-positive and 93 LVI-negative), and patients from center 2 were the external validation cohort (25 LVI-positive and 78 LVI-negative).

### AB-MRI acquisition and pathologic assessment

The AB-MRI protocol included a pre- and early postcontrast phase using a 3D T1 gradient echo with a fat saturation sequence. Detailed AB-MRI parameters are listed in **Table S1**. The early postcontrast images were used for image analysis. In patients with multiple breast cancers, only the largest lesion was selected for analysis.

All of the surgical specimens were examined by two senior pathologists with 16 and 13 years of experience in breast pathology, and the pathologic evaluations are provided in **Supplementary A1**.

### Development of a radiomics signature (RS)

An RS was developed with the following steps: region of interest (ROI) acquisition, feature extraction, feature selection, and model construction. The ROI was manually segmented by a professional radiologist (reader 1, with 11 years of experience in breast imaging). Reader 2 (with 15 years of experience in abdominal imaging) randomly chose 30 patients from the training cohort and performed tumor segmentation for inter-reader agreement analysis. Based on the ROI, 10,402 radiomics features were extracted using the in-house software developed with MATLAB 2016 (Mathworks, Natick, MA, USA), including first-order, shape-based, and texture features. Then, the Mann–Whitney *U* test was used to compare the between-group differences of each radiomics feature in the LVI-positive and LVI-negative groups, and intra-class correlation coefficients (ICCs) were used to evaluate the reproducibility and stability of the radiomics features. The specific process is presented in **Supplementary A2**.

A least absolute shrinkage and selection operator (LASSO) logistic regression was used to build an RS using a linear combination of features based on the selected features. The features with nonzero coefficients were considered valuable predictors for predicting the LVI status, and the tuning parameter was selected by 10-fold cross-validation for the radiomics method. Finally, the output of the RS was labeled as the radiomics score (R-score).

### Development of a transfer learning signature (TLS)

The development of a TLS consisted of two steps: TL features extraction and classification layer training. The first step is the training of feature extraction network. In order to avoid an overfitting of the model, the TL strategy (24) was used to train feature extraction network. The network was first pre-trained using the ImageNet dataset (n = 1.3 million), and the parameters obtained in the pre-training step are taken as the initial parameters of the network. The AB-MRI images were then used to fine-tune the parameters in the network. A total of 11264 TL features were extracted by the network, and the details of feature extraction and selection are presented in **Supplementary A3**.

Based on the TL feature, the differences of the transfer learning signature (TLS) between the LVI-positive and LVI-negative groups were assessed using the Mann–Whitney *U* test. The second step is the classification layer training based on the LASSO logistic regression; the training process was similar to that of RS.

### Visualization of the TLS

For investigating the interpretability of the TLS, the convolutional filter was visualized with gradient-weighted class activation mapping (Grad-CAM) (25), which could produce a localization map highlighting the import regions for classification target. By visualizing the filter, we explored the association between the TL feature and LVI status.

Given an ROI image, each convolutional filter generated a response map showing all the corresponding feature patterns extracted from the lesion. A valuable convolutional filter should have different responses to different types of lesions. Thus, the visualization of the response map for convolutional filters in different lesion groups was helpful to understand the TLS.

### Comparison of the TLS AND RS

We compared the TLS with the RS to comprehensively evaluate the performances of the models.

ROC analysis was performed for the training cohort and external validation cohort to evaluate the diagnostic performance of the TLS and RS. The following parameters were calculated: the area under the curve (AUC), sensitivity, specificity, accuracy, positive predictive value (PPV), and negative predictive value (NPV). The AUCs of the TLS and RS were compared using the DeLong test. In addition, to compare the classification ability of the DLS and RS, the net reclassification index (NRI) and integrated discrimination improvement (IDI) were calculated. Decision curve analysis (DCA) was used to estimate the clinical utility of the TLS and RS. Moreover, a stratified analysis was performed on the menstrual state, molecular subtype, and contrast agent type.

### Statistical analysis

All statistical tests were performed using R3.0.1 (http://www.rproject.org). All radiomics features were extracted with Matlab 2016, and the TL features were extracted with Python 3.6. LASSO was performed using the “glmnet” package, and the ROC curve analysis was performed using the “pROC”. Clinicopathologic characteristics between the LVI-positive and LVI-negative groups were compared using chi-squared test or Mann–Whitney *U* test. p Values <0.05 were considered indicative of a statistically significant difference.

## Results

### Clinicopathologic characteristics

As shown in **Table 1**, in the training and validation cohorts, only the pathological size of the invasive component and sentinel lymph node status were statistically different between the LVI-positive and LVI-negative groups (all p < 0.01), while other characteristics showed no significant difference (p = 0.072–0.876).

**Table 1:** Clinicopathologic characteristics.

	Training cohort (n = 130)			Validation cohort (n = 103)				
	LVI-positive (n = 37)	LVI-negative (n = 93)	p	LVI-positive (n = 25)		LVI-negative (n = 78)		p
Clinical characteristics								
Age (years)			0.103					0.072
≤ 40	14 (37.8%)	22 (23.7%)		10 (40.0%)		17 (21.8%)		
> 40	23 (62.2%)	71 (76.3%)		15 (60.0%)		61 (78.2%)		
Tumor location			0.714					0.876
Upper-outer quadrant	13 (35.1%)	35 (37.6%)		10 (40.0%)		28 (35.9%)		
Upper-inner quadrant	6 (16.2%)	18 (19.4%)		5 (20.0%)		14 (17.9%)		
Lower-outer quadrant	8 (21.6%)	15 (16.1%)		3 (12.0%)		15 (19.2%)		
Lower-inner quadrant	5 (13.5%)	18 (19.4%)		5 (20.0%)		12 (15.4%)		
Central area	5 (13.5%)	7 (7.5%)		2 (8.0%)		9 (11.5%)		
MRI features								
Lesion type			0.362				0.457	
Mass	33 (89.2%)	77 (82.8%)		23 (92.0%)		65 (83.3%)		
NME	4 (10.8%)	16 (17.2%)		2 (8.0%)		13 (16.7%)		
Internal enhancement			0.801					0.577
Homogeneous	2 (5.4%)	8 (8.6%)		1 (4.0%)		8 (10.3%)		
Not homogeneous	35 (94.6%)	85 (91.4%)		24 (96.0%)		70 (89.7%)		
Mass shape			0.604				0.653	
Round or oval	6 (18.2%)	11 (14.3%)		2 (8.7%)		10 (15.4%)		
Irregular	27 (81.8%)	66 (85.7%)		21 (91.3%)		55 (84.6%)		
Mass margin			0.851					0.730
Circumscribed	5 (15.2%)	9 (11.7%)		3 (13.0%)		5 (7.7%)		
Not circumscribed	28 (84.8%)	68 (88.3%)		20 (87.0%)		60 (92.3%)		
Tumor size on MRI, cm (mean ± SD)	3.1 ± 1.0	2.6 ± 0.9	0.147	2.9 ± 0.9		2.5 ± 0.7		0.202
Pathological characteristics								
Pathological size of the invasive component, cm (mean ± S.D.)	2.7 ± 1.0	1.8 ± 0.8	<0.001*	2.4 ± 1.0		1.7 ± 0.9		<0.001*
Sentinel lymph node status			<0.001*				<0.001*	
Positive	23 (62.2%)	13 (14.0%)		18 (72.0%)		9 (11.5%)		
Negative	14 (37.8%)	80 (86.0%)		7 (28.0%)		69 (88.5%)		
Histological grade			0.423				0.656	
I	3 (8.1%)	9 (9.7%)		1 (4.0%)		6 (7.7%)		
II	21 (56.8%)	62 (66.7%)		15 (60.0%)		50 (64.1%)		
III	13 (35.1%)	22 (23.6%)		9 (36.0%)		22 (28.2%)		
Molecular subtype			0.186				0.198	
Luminal A	13 (35.1%)	46 (49.4%)		8 (32.0%)		30 (38.5%)		
Luminal B	9 (24.3%)	27 (29.0%)		7 (28.0%)		33 (42.3%)		
HER2 positive	8 (21.6%)	10 (10.8%)		5 (20.0%)		8 (10.3%)		
Triple negative	7 (18.9%)	10 (10.8%)		5 (20.0%)		7 (8.9%)		

*p < 0.05.

LVI, lymphovascular invasion; NME, non-mass enhancement.

### Performance of the RS

**Figure 2:**
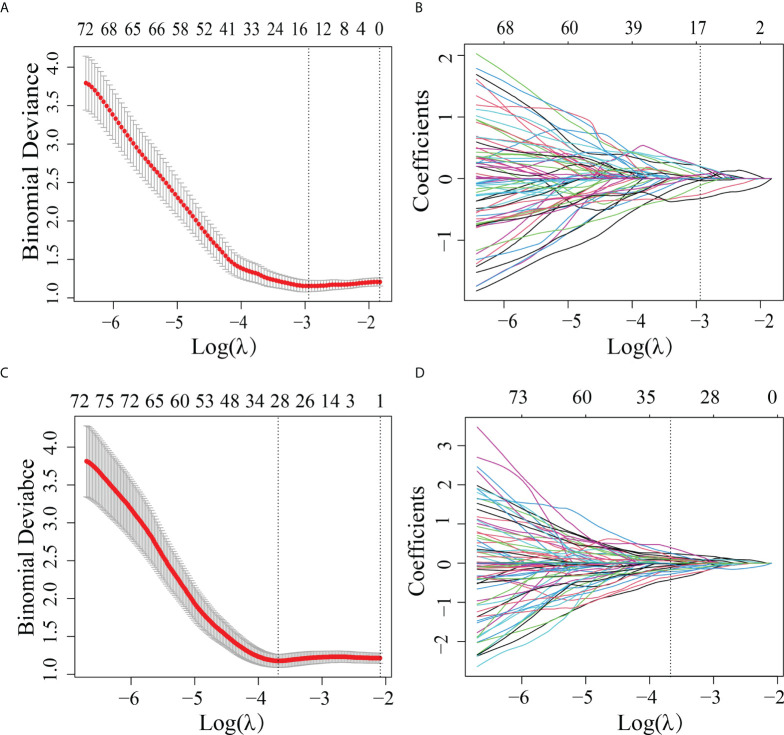
Features selected by the LASSO method. **(A)** Adjustment (λ) selection by 10-fold cross-validation for the radiomics method. Binomial deviance (y-axis) was plotted log(λ) (x-axis). The dotted lines were drawn at the optimal value of λ, where the model provided its best fit. The optimal values of λ and log(λ) were 0.0503 and -2.990, respectively. **(B)** Radiomics feature characteristics of the LASSO coefficient curve. The dashed vertical line was defined with the optimal λ, where 14 optimal radiomics features with nonzero coefficients are indicated. **(C)** Adjustment (λ) selection by 10-fold cross-validation for the transfer learning method. Binomial deviance (y-axis) was plotted log(λ)(x-axis). The dotted lines were drawn at the optimal value of λ, where the model provided its best fit. The optimal values of λ and log(λ) were 0.0241 and -3.730, respectively. **(D)** Transfer learning feature characteristics of the LASSO coefficient curve. The dashed vertical line was defined with the optimal λ, where 28 optimal transfer learning features with nonzero coefficients are indicated.

A total of 2,994 features with a significant difference (p < 0.05) and intraclass correlation coefficient (ICC) values greater than 0.75 were used in the LASSO logistic regression. In the LASSO logistic regression, 14 features with nonzero coefficients (**Figures 2A, B**) were selected as valuable predictors to build the RS by calculating the R-score. The R-score calculation formula and selected features are presented in **Supplementary A4**.

As shown in **Table 2**, the AUC of the RS was 0.850 (95% CI: 0.777–0.906) in the training cohort and 0.726 (95% CI, 0.629–0.809) in the validation cohort.

**Table 2:** A performance summary of RS, NTLS and TLS in the training and validation cohorts for preoperative identification of lymphovascular invasion status in patients with invasive breast cancer.

method		AUC (95% CI)	Sensitivity	Specificity	Accuracy	PPV	NPV
Training cohort	RS	0.850 (0.777-0.906)	0.865 (32/37)	0.710 (66/93)	0.754 (98/130)	0.543 (32/59)	0.930 (66/71)
	TLS	0.911 (0.844-0.954)	0.865 (32/37)	0.839 (78/93)	0.846 (110/130)	0.681 (32/47)	0.940 (78/83)
	NTLS	0.748 (0.631-0.882)	0.838 (31/37)	0.602 (56/93)	0.669 (87/130)	0.456 (31/68)	0.903 (56/62)
External validation cohort	RS	0.726 (0.629-0.809)	0.680 (17/25)	0.846 (66/78)	0.806 (83/103)	0.586 (17/29)	0.892 (66/74)
	TLS	0.852 (0.769-0.915)	0.760 (19/25)	0.846 (66/78)	0.825 (85/103)	0.613 (19/31)	0.917 (66/72)
	NTLS	0.614 (0.552-0.731)	0.800 (20/25)	0.500 (39/78)	0.573 (59/103)	0.339 (20/59)	0.886 (39/44)

TLS, Transfer learning signature; NTLS, Non-transfer learning signature; RS, radiomics signature; AUC, area under curve; PPV, positive predictive value; NPV, negative predictive value; CI, confidence interval.

### Performance of the TLS

In order to differentiate the LVI-positive and LVI-negative groups in the training cohort, 2,907 features were selected according to the Mann–Whitney *U* test with p < 0.05. With LASSO logistic regression, 28 TL features with nonzero coefficients (**Figures 2C, D**) were selected as valuable predictors to build the LVI status-related TLS to calculate the TL-score. The TL-score calculation formula and the selected deep learning features are presented in **Supplementary A5**.

**Table 2** shows that the AUC of the TLS was 0.911 (95% CI: 0.844–954) in the training cohort and 0.852 (95% CI, 0.769–0.915) in the validation cohort.

### Interpretability of the TLS

**Figure 3:**
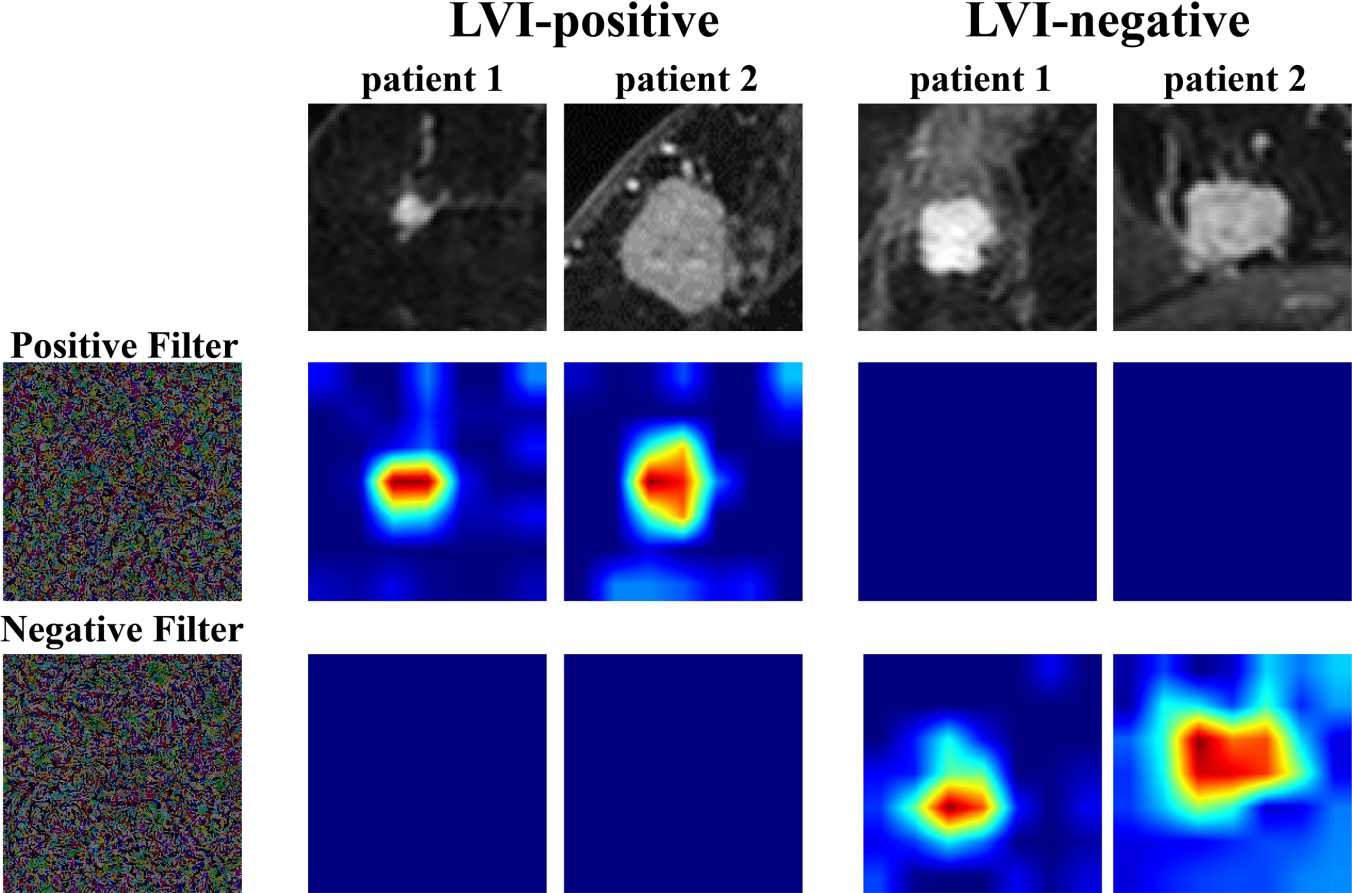
Attention map visualization of LVI-positive and LVI-negative lesions. The first row shows the first postcontrast images from two LVI-positive lesions and two LVI-negative lesions. The second and third rows show the attention maps of the input tumor images. The positive filter has a strong response to LVI-positive lesions, and the negative filter has a strong response to LVI-negative lesions. LVI, lymphovascular invasion.

In order to further understand the association between TL features and LVI status, we extracted two filters, including a positive filter and a negative filter (the first column in **Figure 3**). Based on the filters, the TL model generated an attention map indicating the importance of each part of the lesion. The results showed that the positive filter had strong responses to LVI-positive lesions and weak responses to those that were LVI-negative. Similarly, the negative filter had strong responses to LVI-negative lesions and was nearly shut down in those that were LVI-positive.

### Comparison of the TLS AND RS

**Figure 4:**
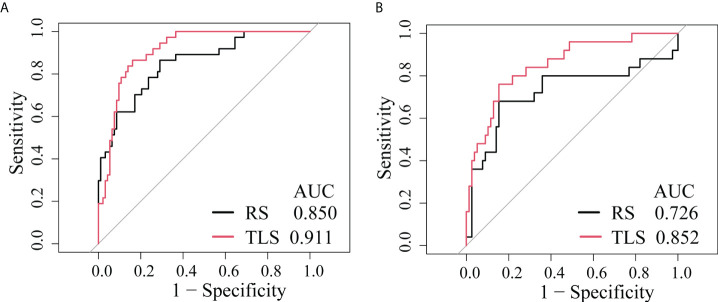
ROC curves of the prediction models. **(A)** Training cohort. **(B)** External validation cohort. RS, radiomics signature; TLS, transfer learning signature.

**Figure 5:**
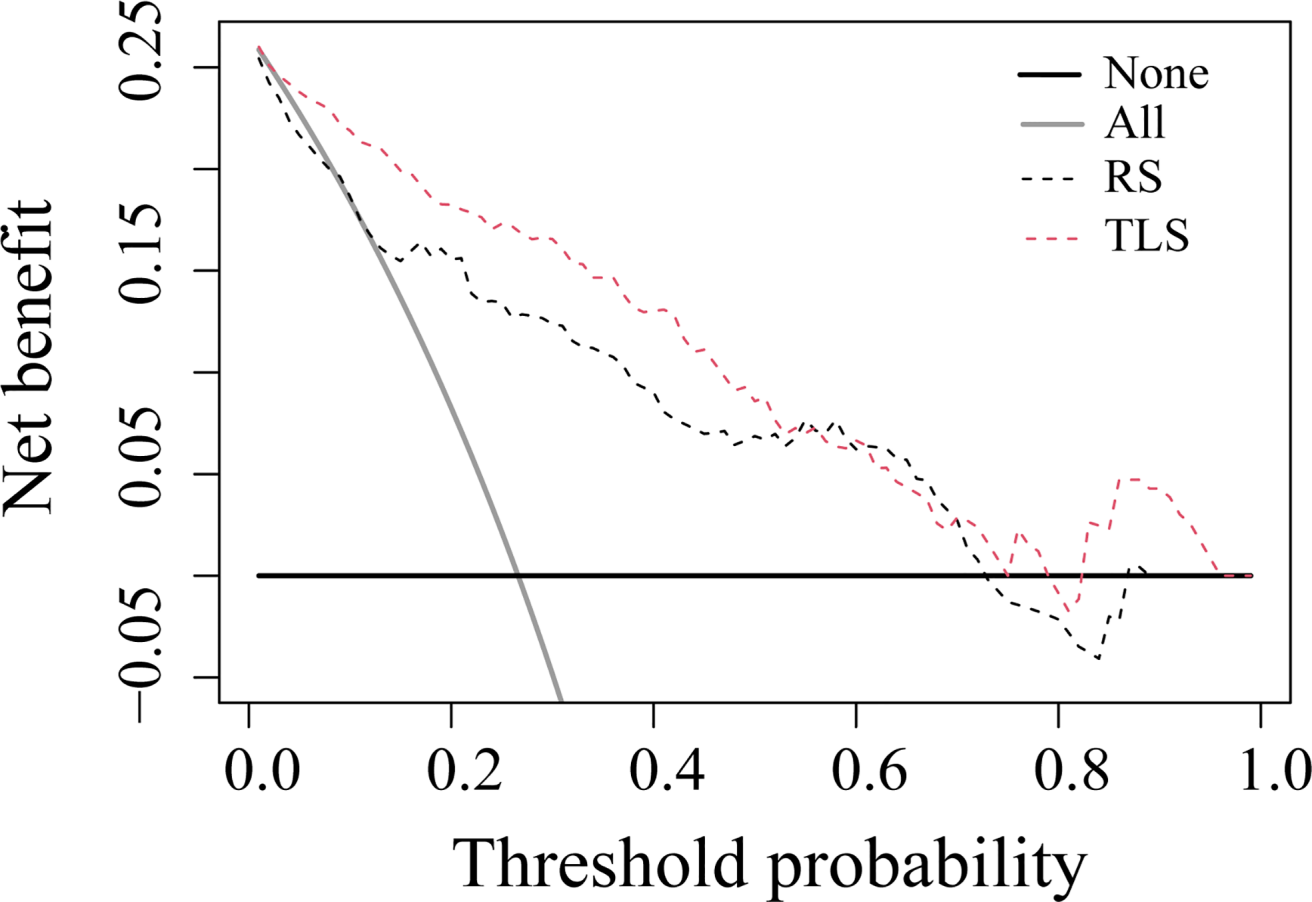
Decision curve analysis for prediction models. The solid gray line represents the assumption that all patients were involved in the LVI-positive group, while the black line represents the assumption that no patients were involved. The threshold probability was the point where the expected benefit of the treatment and treatment avoidance were equal. The results showed that the net benefit of the TLS was greater than that of the RS (range, 0.01–0.95). RS, radiomics signature; TLS, transfer learning signature.

The ROC analysis showed that the TLS yielded a higher AUC value than the RS (0.852 vs. 0.726, p < 0.01; **Table 2** and **Figure 4**). The IDI and NRI demonstrated that, compared with the RS, the TLS achieved better capability in discriminating LVI-positive from LVI-negative lesions (IDI = 0.092, p < 0.001; NRI = 0.554, p < 0.001). DCA illustrated that within the threshold probability range of 0.01 and 0.95, the TLS gained a greater net benefit than the RS (**Figure 5**). Stratified analysis showed that the performance of the TLS was not affected by the menstrual state, molecular subtype, and contrast agent type (all p > 0.05; **Supplementary A6**).

## Discussion

Preoperative prediction of LVI might provide useful information in the management of neoadjuvant chemotherapy and axillary surgery in IBC patients with clinically negative nodes (4–6). As AB-MRI is increasingly applied in breast cancer screening or lesion diagnosis, more and more breast cancers were detected by AB-MRI first. Part of these IBC patients only undergo AB-MRI without the full protocol. Thus, we try to investigate whether LVI status can be assessed simultaneously when IBC is detected on AB-MRI. The current study developed a TLS and an RS based on AB-MRI to predict LVI in IBC patients with clinically negative nodes. Their diagnostic performances were validated and compared in an external cohort. Our results showed that the TLS had a better discriminating ability between LVI-positive and LVI-negative lesions than the RS. The satisfied diagnostic performance suggested that AB-MRI not only could detect breast cancer but could also be effective in predicting LVI status simultaneously when transfer learning algorithm was introduced.

In our study, two clinicopathologic characteristics, i.e., pathological size of the invasive component and sentinel lymph node metastasis, were significantly different between LVI-positive and LVI-negative lesions. These two characteristics were determined after surgery and provided no preoperative value. Therefore, they were not incorporated into the radiomics and TL model for the preoperative prediction of LVI. Notably, sentinel lymph node metastasis was more frequent in the LVI-positive group, which indicated that a sentinel lymph node biopsy could not be omitted in LVI-positive patients despite clinically negative nodes. Accordingly, preoperative identification of the LVI status might be helpful in clinical decision-making with sentinel lymph node biopsy (26).

Previous studies have investigated preoperative LVI prediction with various imaging modalities. Digital mammography was reported to be insufficient in predicting LVI (27). Ultrasound, especially the elastic heterogeneity value achieved high sensitivity but mediocre specificity (28). MRI is the most common modality used for LVI assessment in breast cancer. Multiparametric MRI based radiomics has yielded satisfied diagnostic performance (29). However, the time consumption and high cost hinder the broader use of the multiparametric MRI. In this case, AB-MRI based radiomics was initially applied to LVI evaluation in breast cancer, while the diagnostic performance was only moderate (16). The RS in that study yielded a similar AUC value to the present study in the validation cohort (AUC: 0.752 vs. 0.726). The results of the previous and current studies indicated the feasibility of AB-MRI-based radiomics for LVI evaluation. However, the performance of RS was slightly below satisfaction for clinical use. The possible reason is that the radiomic features extracted from a fixed set cannot completely and accurately reflect the subtle differences between LVI-positive and LVI-negative lesions. In addition, manual lesion delineation is extremely labor-intensive and time-consuming, limiting the clinical application of radiomics. Accordingly, an advanced machine learning approach is needed to improve diagnostic accuracy and reduce image processing complexity.

In contrast, the TL algorithm is a candidate method to automatically learn to capture useful features on images without manual tumor segmentation (18, 19). It has become a promising tool in the studies of breast imaging, such as breast cancer screening (30), cancer risk stratification (31), lesion classification (32), and axillary lymph node metastasis predictions (33). Thus, we applied a TLS in LVI prediction. As expected, compared with the RS, the TLS improved the diagnostic performance significantly in the validation cohort (AUC: 0.726 vs. 0.852). Similarly, performance improvement with TL was also observed in other breast imaging studies, such as breast lesion classification (17) and lymph node metastasis prediction (34). The results suggested that TLS can mine more relevant image features to reflect the high-dimensional association between images and clinical issues. These image features were generated and extracted using multiple layers of self-learning units in the TL method. They were different from visual subjective findings or radiomic features. Furthermore, in order to further verify the effectiveness of the proposed method, we construct a TL model (BotELM) based on the bottleneck transformer network (BotNet) and extreme learning machine (ELM), we validated it on external validation cohort (**Supplementary Table S1**). The experimental results again prove that the TL model BotELM improved the diagnostic performance significantly based on AB-MRI compared with the RS (AUC: 0.760 vs. 0.726), while the AUC of BotELM is lower than TLS in external validation cohort.

Moreover, we visualized the TLS *via* a convolutional filter visualization technique to further understand the instinctual relationship between deep learning features and LVI. For the positive filter, the attention map illustrated that the tumor and peritumor areas were two high-response locations in LVI-positive lesions, while they were not in the LVI-negative lesions. In contrast, the negative filter had strong responses to LVI-negative lesions and was nearly shut down in lesions that were LVI-positive. To some extent, this supported the effectiveness of the model.

However, it is important to point out that the performance of CNN without transfer learning (NTLS) was poor in the validation cohort (**Table 2**), even compared with the traditional RS (AUC: 0.614 vs. 0.726). The main reason for this result is that the CNN method works well only when enough labeled training data is available (35), while the labeled training data is small in the clinical practice. Thus, in order to ameliorate the effect of small labeled training data, the use of TL strategy is ubiquitous. How different TL strategies affect the performance of CNN is our next research content.

There were several limitations in our study. First, the sample size was relatively small, especially the training cohort, which did not meet the traditional CNN modeling requirement. To overcome this shortage, we pretrained the network with TL. The model based on a transfer learning strategy can avoid overfitting in a training dataset, which reduces the amount of data required for modeling. Nevertheless, the generalization of a TLS still needs to be validated in other centers. Second, multiparametric MRI-based TLS performance was not investigated as the purpose of the study is to investigate whether LVI status can be assessed simultaneously when IBC is detected on AB-MRI. The included patients only underwent AB-MRI for breast cancer screening or lesion diagnosis. However, full diagnostic protocol should be compared with an AB-MRI for LVI prediction in prospective studies. Finally, our study was based on the construction of a two-dimensional slice feature model, and the performance of the three-dimensional features remains to be further studied.

In summary, the TLS was superior to the RS for LVI prediction in IBC patients with clinically negative nodes. The proposed AB-MRI-based TLS could potentially serve as an easy-to-access and easy-to-use approach to assist individual breast cancer treatments.

## Data availability statement

The original contributions presented in the study are included in the article/**Supplementary Material**. Further inquiries can be directed to the corresponding authors.

## Ethics statement

The studies involving human participants were reviewed and approved by Jiangmen Central Hospital and Nanfang Hospital. The patients/participants provided their written informed consent to participate in this study.

## Author contributions

WL and LZ designed the research. XL, XC, and RL collected the data. BF, YL, YC, HZ, and TY contributed data analysis tools and performed the analysis; BF also acquired the funding. ZL and BF wrote the paper. WL, EC, and LZ supervised the study. All authors contributed to the article and approved the submitted version.

## Funding

This work was supported by the National Natural Science Foundation of China (81960324, 62176104, and 61876064), the National Natural Science Foundation of Guangxi (2021GXNSFAA075037), the Guilin University of Aerospace Technology Foundation (XJ21KT17), the Guangdong Basic and Applied Basic Research Foundation (2019A1515011773), the Pearl River S&T Nova Program of Guangzhou under Grant (201906010043), Elite Young Scholars Program of Jiangmen Central Hospital (J201904), and Medical Scientific Research Foundation of Guangdong Province of China (A2020622).

## Conflict of interest

The authors declare that the research was conducted in the absence of any commercial or financial relationships that could be construed as a potential conflict of interest.

The reviewer WL declared a shared affiliation with several of the authors, TY and LZ, to the handling editor at time of review.

## Publisher’s note

All claims expressed in this article are solely those of the authors and do not necessarily represent those of their affiliated organizations, or those of the publisher, the editors and the reviewers. Any product that may be evaluated in this article, or claim that may be made by its manufacturer, is not guaranteed or endorsed by the publisher.

## Abbreviations

LVI, lymphovascular invasion; IBC, invasive breast cancer; AB-MRI, abbreviated breast magnetic resonance imaging; TLS, transfer learning signature; LASSO, least absolute shrinkage selection operator; RS, radiomics signature; CI, confidence interval; NRI, net reclassification improvement; IDI, integrated discrimination improvement.
