# Supplementary material for: Predicting lymphovascular invasion in clinically node-negative breast cancer detected by abbreviated magnetic resonance imaging: Transfer learning vs. radiomics
Source: Front Oncol. 2022 Sep 15;12:890659. doi: 10.3389/fonc.2022.890659 (PMC9520481; doi:10.3389/fonc.2022.890659)
Supplement: Supplementary file 1 [file DataSheet_1.doc]

**Supplementary A1: The inclusion and exclusion criteria of the patient**

Inclusion criteria:

(a) underwent AB-MRI and have enhanced lesions on MR image;

(b) lesions diagnosed as invasive ductal carcinoma based on pathologic evaluation of surgical specimens;

(c) time interval between surgery (mastectomy or lumpectomy) and MRI examination: < 2 weeks.

Exclusion criteria:

(1) biopsy performed before AB-MRI (n=58);

(2) received neoadjuvant chemotherapy (n=30) or radiotherapy (n=55) before MRI examination;

(3) node-positive diagnosed on preoperative ultrasonography (n=37): rounded hypoechoic node; complete or partial effacement of the fatty hilum; focal or diffuse cortical thickening (≥5 mm); complete or partial replacement of the node with an ill-defined or irregular mass; extra-hilar blood vessel flow on color Doppler images; or microcalcifications in the node [1];

(4) MR image with obvious artifacts (n=15);

(5) diagnosed as benign tumors, high-risk lesions, pure ductal carcinoma in situ or special types of invasive breast carcinoma at final pathologic evaluation (n=69)

**Supplementary A2: Pathologic evaluation**

Surgical resection specimens were evaluated by two pathologists with 16 and 13 years of experience in breast pathology. Specimens in which tumor cells were detected within the lymphatic or vascular space in the peripheral area of the tumor were classified as LVI-positive. If the evaluation of HE-stained sections was uncertain, immunohistochemical staining such as D2-40 and CD31 were performed. The histopathological type, histological grade, molecular subtype and pathological size of the invasive component were also assessed.

All enrolled patients received sentinel lymph node biopsy. The status of sentinel lymph node was evaluated.

**Supplementary A3: The tumor segmentation, feature extraction and feature selection**

**The tumor segmentation:** The region of interest (ROI) was manually segmented by a professional radiologist (reader 1, with 11 years of experience in breast imaging) using our in-house software developed with MATLAB 2016 (Mathworks, Natick, MA, USA). Contour lines of the ROI were drawn along the boundaries of the tumor on each axial image, avoiding the adjacent air. Subsequently, reconstruction of the whole tumor volume was performed using the identified axial images. In order to enhance the robustness and repeatability of extraction of the radiomics features, wavelet bandpass filtering, isotropic resampling, and grayscale discretization were applied while reconstructing the whole tumor volume.

**Feature extraction:** Features were extracted using the in-house software developed with MATLAB 2016 (Mathworks, Natick, MA, USA). The extracted radiomics features were standardized by the z-score. 10402 quantitative imaging features were extracted, based on the original image and its corresponding filtered image, and included the features from the categories of first order statistics (n=78), shape (n=4), Global (n=720), gray-level co-occurrence matrix (GLCM, n=2160), gray-level run-length matrix (GLRM, n=3120), gray-level size-zone matrix (GLSZM, n=3120) and neighborhood gray tone difference matrix (NGTDM, n=1200) [1].

**Feature selection:** We used the Mann–Whitney U test to compare the between-group differences of each radiomics feature in the LVI-positive and LVI-negative. Intraclass correlation coefficients (ICCs) were used to evaluate the reproducibility and stability of the radiomics features. Reader 1 performed the segmentation on all of the patients. Reader 2 (with 15 years of experience in breast imaging) randomly chose 30 patients from the training cohort and performed tumor segmentation for inter-reader agreement analysis. The radiomics features with significant difference (P<0.05) and ICC values greater than 0.75 were used for least absolute shrinkage and selection operator (LASSO) logistic regression.

**Supplementary A4: The transfer learning features extraction and selection process**

**The tumor segmentation:** Before the extraction process of features, region of interest (ROI) for the lesions were manually segmented by the same two radiologists (reader 1 and reader 2) using MATLAB 2016 software (Mathwork Natick, MA, USA). Since the input of the deep learning model is rectangular images containing the entire ROI lesions, all slices images of the tumor lesion were resized to 224 × 224 size images. Moreover, three slices of the same rectangular frame were cut consecutively from the tumor lesion images, and then the tumor ROIs of each lesion were extracted and composed into a three-channel image. Finally, the tumor images with 224 × 224 × 3 size images were standardized using the z-score method, and then fed into the DensNet121 network.

**Feature extraction:** The transfer learning features extraction was implemented using the Keras toolkit and Python 3.7. Transfer learning features were extracted by convolution neural network based on DensNet121 network, which is based on the Densenet121 network. The network has four dense blocks that each has an equal number of layers. Before entering the first dense block, a convolution with 7×7 is performed on the input images. For convolutional layers with kernel size 3×3, each side of the inputs is zero-padded by one pixel to keep the feature-map size fixed. We use 1×1 convolution followed by 2×2 average pooling as transition layers between two contiguous dense blocks. At the end of the last dense block, a global average pooling is performed and then a softmax classifier is attached. The outputs of the convolutional layer generally were the transfer learning features.

Firstly, in order to avoid-fitting of the model, the transfer learning strategy [2] was used to train DenseNet121 model with ImageNet dataset (n = 1.3 million). Moreover, to increase the robustness of the prediction, all the MRI slices of the lesions were fed into the deep learning model. The deep learning features were calculated as the average probability from all slices. Finally, the transfer learning features extracted from the network were designated as the outputs of the CNN’s hidden layers for transfer learning features, and 11264 transfer learning features referred to the output of the convolution layer. The extraction process was performed is shown in **Figure S1**.

**Feature selection:** Differences of the transfer learning signature (TLS) between the LVI-positive and LVI-negative were assessed by the Mann–Whitney U test. To avoid over-fitting, the minimum-redundancy maximum-relevance algorithm was used to reduce feature dimensions by calculating feature redundancy and the first 15% features with minimum redundancy were selected for TLS construction.

**Supplementary A5: Radiomics score formula**

For the radiomics signature, the radiomics score (R-score) calculation formula by LASSO method is as follows:

R-score=-1.0227+0.05911×feature1_(R=0.50,Scale=0.8,Quant.algo=Equal,Ng=64,GLSZM-LZHGE)+0.20209×feature2_(R=0.50,Scale=1.2,Quant.algo=Equal,Ng=16,GLSZM-LZLGE)-0.32525×feature3_(R=0.50,Scale=1.2,Quant.algo=Lloyd,Ng=32,NGTDM-Complexity)+0.03294×feature4_(R=0.67,Scale=0.8,Quant.algo=Lloyd,Ng=16,GLSZM-LZLGE)-0.02987×feature5_(R=0.67,Scale=1.2,Quant.algo=Lloyd,Ng=32,GLSZM-ZP)-0.02624×feature6_(R=0.67,Scale=1.5,Quant.algo=Lloyd,Ng=32,NGTDM-Complexity)+0.09994×feature7_(R=1.00,Scale=1,Quant.algo=Equal,Ng=64,GLSZM-LZHGE)+0.0082×feature8_(R=1.00,Scale=1,Quant.algo=Lloyd,Ng=32,GLCM-Variance)+0.06394×feature9_(R=1.00,Scale=1.2,Quant.algo=Equal,Ng=64,GLSZM-GLV)+0.19649×feature10_(R=1.50,Scale=pixelW,Quant.algo=Lloyd,Ng=8,GLCM-Variance)+0.02955×feature11_(R=1.50,Scale=0.8,Quant.algo=Lloyd,Ng=16,GLSZM-SZLGE)+0.20061×feature12_(R=1.50,Scale=2,Quant.algo=Equal,Ng=32,GLRLM-SRLGE)

-0.11376×feature13_(R=2.00,Scale=2,Quant.algo=Lloyd,Ng=16,GLSZM-SZLGE)-0.02483×feature14_SUVmax

**Supplementary A6: Transfer learning score formula**

For the transfer learning signature, the transfer learning score (TL-score) calculation formula by LASSO method is as follows:

TL-score=-1.8443-0.01349×feature1_conv3_2_x2_27+0.10751×feature2_conv3_5_x1_100+0.40454×feature3_conv3_8_x1_13-0.17317×feature4_conv4_2_x1_36-0.21915×fetaure5_conv4_8_x1_118-0.35141×feature6_conv4_13_x1_11-0.02806×feature7_conv4_15_x2_20+0.09751×feature8_conv4_16_x1_29-0.16972×feature9_conv_blkmat_224+0.17416×feature10_conv4_blkmat_261-0.08372×feature11_conv5_4_x1_63-0.1316×feature12_conv5_4_x1_88-0.01645×feature13_conv5_4_x1_100+0.12848×feature14_conv5_7_x1_120+0.06797×feature15_conv5_9_x1_113-0.42889×feature16_conv5_9_x2_6-0.28368×feature17_conv5_10_x1_124+0.16585×feature18_conv5_13_x1_111+0.00068×feature19_conv5_13_x1_127-0.00021×feature20_conv5_13_x2_1-0.05999×feature21_conv5_15_x1_88+0.09643×feature22_conv5_blk_bn_696 +0.24481×feature_conv5_blk_bn_789+0.08896×feature24_conv5_blk_bn_850 +0.057745×feature25_conv5_blk_bn_911+0.13113×feature26_conv5_blk_bn_927-0.16114×feature27_conv5_blk_bn_937 +0.2641×feature28_conv5_blk_bn_955.


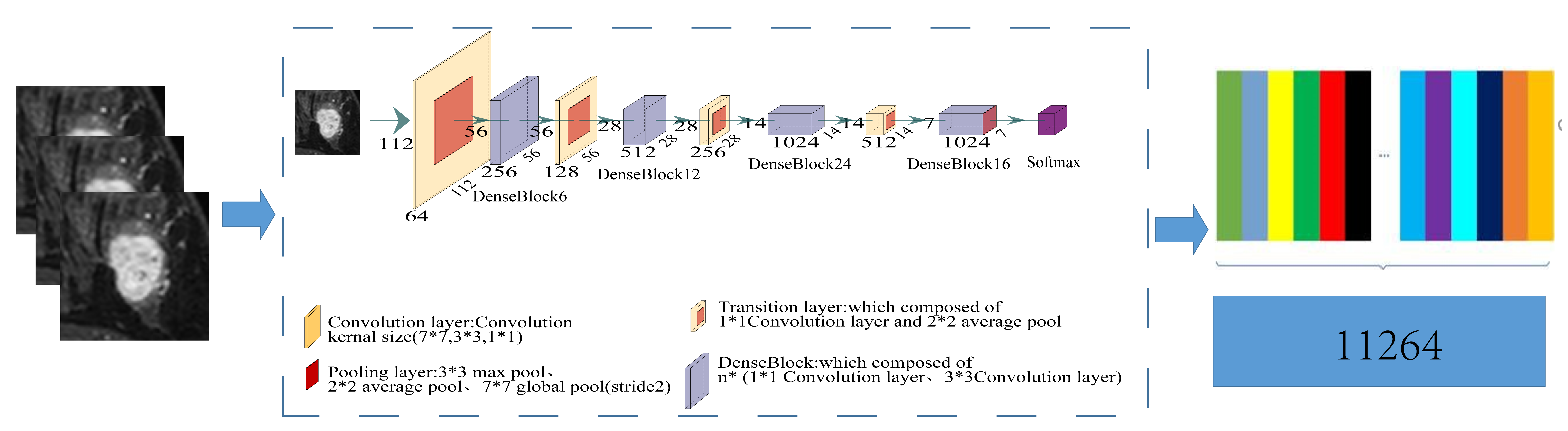


FIGURE S1. The transfer learning features extraction process.

**Supplementary A7: Stratified analysis**

In order to evaluate robustness of the TLS, we performed stratified analysis based on menstrual state, molecular subtype and contrast agent type. We used the ROC curve and AUC to assess the performance of TLS. The results showed that TLS was not influenced by these factors.

1. Stratified analysis of menstrual state

The AUCs of the menstrual state (Premenopausal and Postmenopausal) were 0.913 and 0.816 **(FIGURE. S2a)**, respectively. The p values of premenopausal group compared with postmenopausal was 0.130 by Delong test.

1. Stratified analysis of the molecular subtype

The AUCs of the molecular subtype (triple negative and other subtypes) were 0.856 and 0.898 **(FIGURE. S2b)**, respectively. The p values of triple negative group compared with the other subtypes group was 0.523 by Delong test.

1. Stratified analysis of the contrast agent type

The AUCs of the contrast agent type (gadopentate dimeglumine and gadobutrol) were 0.883 and 0.714 **(FIGURE. S2c)**, respectively. The p values of gadopentate dimeglumine group compared with the gadobutrol group was 0.107 by Delong test.


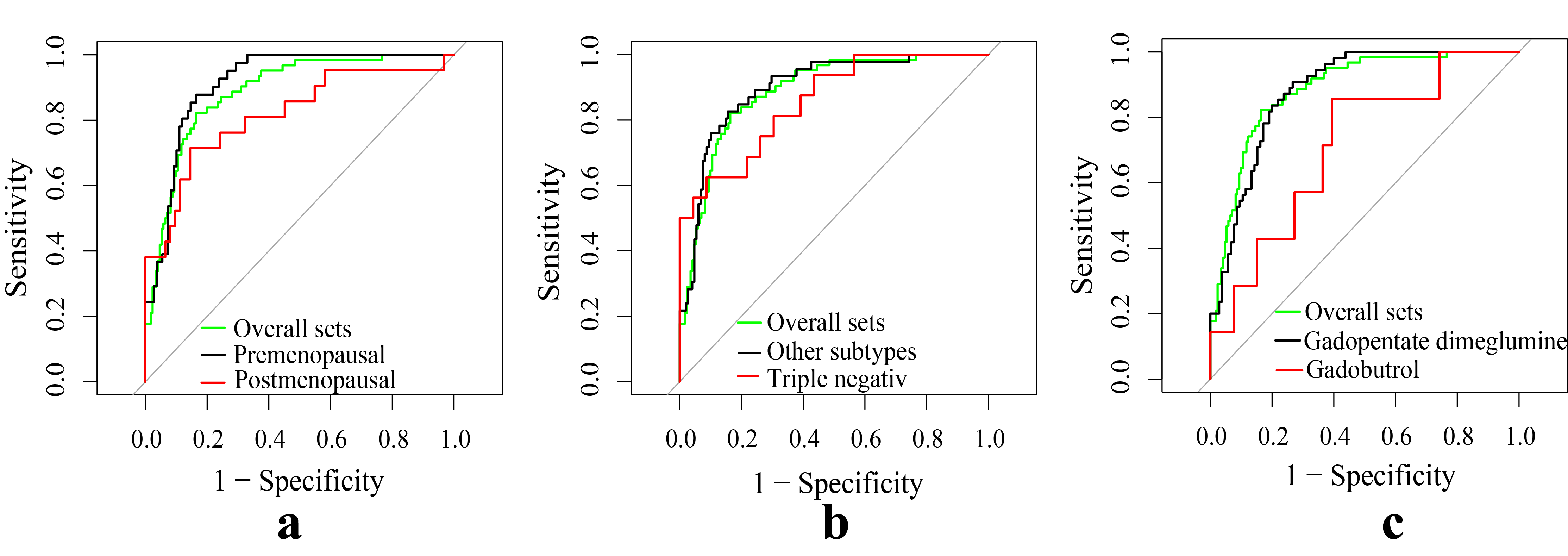


Fig. S2. The ROCs with radiomics nomogram score for each group stratified by the menstrual state (**a**), molecular subtype (**b**) and contrast agent type (**c**).

**Supplementary A8: The development of BotELM model**

Based on the bottleneck transformer network (BotNet) and extreme learning machine (ELM), we construct a transfer learning model BotELM. First, transfer learning features were extracted by convolution neural network based on BotNet. The network takes residual network (ResNet18) [3]as the main backbone, and then replaces the last convolutional layer of the network with the multi-head attention mechanism. Transfer learning features were extracted by convolution neural network BotNet.

Second, differences of the BotELM between the LVI-positive and LVI-negative were assessed by the Mann–Whitney U test. To avoid over-fitting, the minimum-redundancy maximum-relevance algorithm was used to reduce feature dimensions by calculating feature redundancy and the first 15% features with minimum redundancy were selected for BotELM construction.

Finally, ELM was used to build an classification model using the selected features.

TABLE S1 A performance summary of RS, TLS and BotELM in the training and validation cohorts

| method | | AUC  (95% CI) | Sensitivity | Specificity | Accuracy | PPV | NPV |
| --- | --- | --- | --- | --- | --- | --- | --- |
| Training cohort | RS | 0.850  (0.777-0.906) | 0.865  (32/37) | 0.710  (66/93) | 0.754  (98/130) | 0.543  (32/59) | 0.930  (66/71) |
|  | TLS | 0.911  (0.844-0.954) | 0.865  (32/37) | 0.839  (78/93) | 0.846  (110/130) | 0.681  (32/47) | 0.940  (78/83) |
|  | BotELM | 0.820  (0.743-0.882) | 0.703  (26/37) | 0.806  (75/93) | 0.777  (101/130) | 0.591  (26/44) | 0.872  (75/86) |
| External validation cohort | RS | 0.726  (0.629-0.809) | 0.680  (17/25) | 0.846  (66/78) | 0.806  (83/103) | 0.586  (17/29) | 0.892  (66/74) |
|  | TLS | 0.852  (0.769-0.915) | 0.760  (19/25) | 0.846  (66/78) | 0.825  (85/103) | 0.613  (19/31) | 0.917  (66/72) |
|  | BotELM | 0.760  (0.665-0.838) | 0.640  (16/25) | 0.756  (59/78) | 0.728  (75/103) | 0.457  (16/35) | 0.868  (59/68) |

From these results in Table 1, we can obtain the following insightful observations.

1. Compared with RS, BotELM has better performance and the AUC is 0.760 in the external validation cohort. BotELM, which is based on the transfer learning framework and abbreviated breast magnetic resonance imaging (AB-MRI), can extract specific features related to tumors in higher dimensions.
2. The feature extracting network BotNet takes residual network (ResNet18) as the main backbone, and then replaces the last convolutional layer of the network with the multi-head attention mechanism. Compared with TLS, the multi-head attention mechanism may be more effective based on more samples, thus the AUC of BotNet is lower than TLS in external validation cohort.
3. The proposed TLS method based on AB-MRI obtains the best results on the LVI prediction in patients with clinically node-negative IBC classification task, verifying its effectiveness and superiority.

TABLE 1 A performance summary of RS, TLS and BotELM in the training and validation cohorts

**Reference:**

1. Feng B, Chen XM, Chen YH et al (2020). Solitary solid pulmonary nodules: a CT-based deep learning nomogram helps differentiate tuberculosis granulomas from lung adenocarcinomas. Eur Radiol. 30(12):6497-6507.
2. Annegreet V O, M. Arfan I et, al (2015). Transfer learning improves supervised image segmentation across imaging protocols. IEEE Trans. Med. Imaging. 34(5):1018–1030.
3. He K, Zhang X, Ren S, et al. Deep residual learning for image recognition[C]//IEEE Conference on Computer Vision And Pattern Recognition. 2016: 770-778.

**Supplementary A8: TABLE S1:** AB-MRI parameters

| Parameters | Center 1 | Center 2 |
| --- | --- | --- |
| MRI scanner | Ingenia (Philips Healthcare) | uMR780 (United Imaging Healthcare) |
| Field strength | 3.0 T | 3.0 T |
| Breast coil | seven-channel | ten-channel |
| TR/TE | 3.9/2.0 ms | 4.5/1.7 ms |
| Flip angle | 12° | 10° |
| Field of view^†^ | 280 - 320 mm | 280 - 320 mm |
| Image matrix | 280 × 340 | 280 × 340 |
| Section thickness/gap | 1.0/0 mm | 1.0/0 mm |
| Acquisition time/phase^†^ | 66-73 s | 59-65 s |
| NSA | 1 | 1 |
| Contrast agent  type | gadopentate dimeglumine (Magnevist, Bayer) or gadobutrol (Gadovist, Bayer) | gadopentate dimeglumine (Magnevist, Bayer) or gadobutrol (Gadovist, Bayer) |
| Contrast agent dosage | 0.1 mmol/kg | 0.1 mmol/kg |
| Contrast agent infused rate | 2.0 ml/s | 2.0 ml/s |
| post-contrast phase  interval time | 60 s after injection of  contrast agent | 60 s after injection of  contrast agent |

*T*, tesla; *TR*, time of repetition; *TE*, time of echo; *NSA*, number of signal average.

^†^ Field of view and acquisition time/phase depend on breast size.
